# SiPM Developments for the Time-Of-Propagation Detector of the Belle II Experiment

**DOI:** 10.3390/s25134018

**Published:** 2025-06-27

**Authors:** Flavio Dal Corso, Jakub Kandra, Roberto Stroili, Ezio Torassa

**Affiliations:** 1 INFN Sezione di Padova, I-35131 Padova, Italy; flavio.dalcorso@pd.infn.it (F.D.C.); jakub.kandra@pd.infn.it (J.K.); ezio.torassa@pd.infn.it (E.T.); 2 Dipartimento di Fisica e Astronomia, Università degli Studi di Padova, I-35131 Padova, Italy

**Keywords:** silicon photo-multipliers, radiation hardness, Belle II detector, Time-Of-Propagation

## Abstract

Belle II is a particle physics experiment working at an high luminosity collider within a hard irradiation environment. The Time-Of-Propagation detector, aimed at the charged particle identification, surrounds the Belle II tracking detector on the barrel part. This detector is composed by 16 modules, each module contains a finely fused silica bar, coupled to microchannel plate photomultiplier tube (MCP-PMT) photo-detectors and readout by high-speed electronics. The MCP-PMT lifetime at the nominal collider luminosity is about one year, this is due to the high photon background degrading the quantum efficiency of the photocathode. An alternative to these MCP-PMTs is multi-pixel photon counters (MPPC), known as silicon photomultipliers (SiPM). The SiPMs, in comparison to MCP-PMTs, have a lower cost, higher photon detection efficiency and are unaffected by the presence of a magnetic field, but also have a higher dark count rate that rapidly increases with the integrated neutron flux. The dark count rate can be mitigated by annealing the damaged devices and/or operating them at low temperatures. We tested SiPMs, with different dimensions and pixel sizes from different producers, to study their time resolution (the main constraint that has to satisfy the photon detector) and to understand their behavior and tolerance to radiation. For these studies we irradiated the devices to radiation up to $5\times 10^{11}\;1$ MeV neutrons equivalent (neq) per cm^2^ fluences; we also started studying the effect of annealing on dark count rates. We performed several measurements on these devices, on top of the dark count rate, at different conditions in terms of overvoltage and temperatures. These measurements are: IV-curves, amplitude spectra, time resolution. For the last two measurements we illuminated the devices with a picosecond pulsed laser at very low intensities (with a number of detected photons up to about twenty). We present results mainly on two types of SiPMs. A new SiPM prototype developed in collaboration with FBK with the aim of improving radiation hardness, is expected to be delivered in September 2025.

## 1. Introduction

The Belle II detector and the SuperKEKB collider are operating at KEK laboratory (Tsukuba) since 2018. The Belle II detector has collected an integrated luminosity of more than 500 fb^−1^. In 2024, the SuperKEKB accelerator has achieved the world’s leading peak luminosity, $5.1\times 10^{34}\;{\mathrm{cm}}^{-2}s^{-1}$ for an $e^{+}e^{-}$ collider.

The Time-Of-Propagation detector (TOP) [1,2] measures the position and arrival time of Cherenkov photons produced by charged particles crossing the thin quartz bars surrounding the barrel region of the experiment. The number of photons produced in the radiator is quite small (of the order of twenty) and each photodetector is measuring the arrival time of a single photon; in order to identify the particle crossing the quartz bar the photon arrival time must be measured with a resolution better than 100 ps.

The experiment’s first year’s operation showed a machine background much larger than expected; this has a strong effect on the lifetime of the detector components, especially for the TOP detector, whose photodetectors are subject to degradation of quantum efficiency with the accumulated charge.

During the long shutdown in 2023 (LS1), the Micro-Channel Plate Photomultiplier Tubes (MCP-PMTs) were partially replaced with life-extended Atomic Layer Deposition (ALD) MCP-PMTs, that are more resistant to radiation damage; it will be completed in next long shutdown (LS2) or during a summer shutdown.

According to background simulation, which takes into account Bhabha scattering, two-photon process, beam-gas interactions, Touschek effect and the expected gradual increase of peak luminosity, the life-extended ALD MCP-PMTs should survive and maintain performance without significant degradation until the integrated luminosity of about $10\;{\mathrm{ab}}^{-1}$ is achieved.

It is clear that background levels beyond the current projection will be untolerable for this kind of photodetectors, and, from experience, we know that background projections in general are quite optimistic. For this reason an R&D program started in order to find alternatives to MCP-PMT, one of these options is to use silicon photomultipliers. The impact of this choice is not negligible on the TOP detector as the electronics and mechanical support has to be redesigned. The most impacting factor, though, is coming, as will be shown in this paper, from the fact that the operating temperature of the detector must be well below the ambient temperature. This means taking into account mechanical and thermal stresses and introducing a complicated cooling system that should interest only the photodetectors and probably part of the readout chain.

One of the advantages of SiPMs is that they can operate in magnetic fields without any effect on their performance, but they are quite sensitive to radiation damage, especially the one caused by neutrons, which mainly affects the noise in terms of dark count rates and that is quite important when dealing with single-photon signals, as is the case for the TOP detector. A side effect of the large dark count rate is the worsening of the time resolution given by the distortion of the signal leading edge due to the tail of background signals.

At target instantaneous luminosity of $6\times 10^{35}\;{\mathrm{cm}}^{-2}\cdot s^{-1}$, the expected neutron fluence near the photodetector region is about $2\times 10^{10}\;\mathrm{neq}/{\mathrm{cm}}^{2}$ per year.

The plan is to look beyond the current running conditions and work for the upgrade of the collider and the experiment; in this optic we have to consider a lifetime of the machine/experiment of at least ten years and improvement of the luminosity, that means an integrated fluence of more than $1\times 10^{11}\;\mathrm{neq}/{\mathrm{cm}}^{2}$.

The target of this work is:

- to study the radiation damage on SiPMs and possible techniques to partially mitigate its effect;
- to define the SiPM size;
- to find out the best cell size;
- to determine the working temperature;

In order to get the best performances in terms of time resolution and dark count rate.

Several studies are ongoing to evaluate the possible use of SiPMs as photodetectors in particle detectors subjected to intermediate or high neutron irradiation [3,4,5,6,7,8,9,10,11].

In this work, we studied 32 devices from four different producers: Hamamatsu (Hamamatsu City, Japan), FBK (Trento, Italy), Ketek (Munich, Germany) and OnSemi (Phoenix, AZ, USA). These devices, listed in Table 1, are grouped by 8, as we can test 8 SiPMs in parallel with our setup, and in this paper quite often we will refer to the group names H1, FBK1, MIX1, H2 (see Table 1). In Table 2 the geometrical characteristics of the SiPM models, used in this work, are listed.

All modules have been irradiated at the CN accelerator in LNL INFN laboratory in Legnaro after a first detailed characterization. The irradiation doses are different for different groups and even for single devices in order to study the radiation damage, details on the irradiation will be given in the following.

The devices were also annealed after irradiation in order to understand the possible recovery strategy.

Details of experimental setup, irradiation campaigns and modules are described in Section 2.

Processed data was analyzed offline by software and analysis tools described in Section 3. In Section 4, the obtained results for IV-curves, photon spectra fits, time resolution and dark count measurement are shown for several temperatures collected before and after irradiations and annealings.

The devices were annealed by putting them in an oven with a stable temperature for a continuous time interval.

## 2. Experimental Setup and Irradiation Campaigns

**Figure 1:**
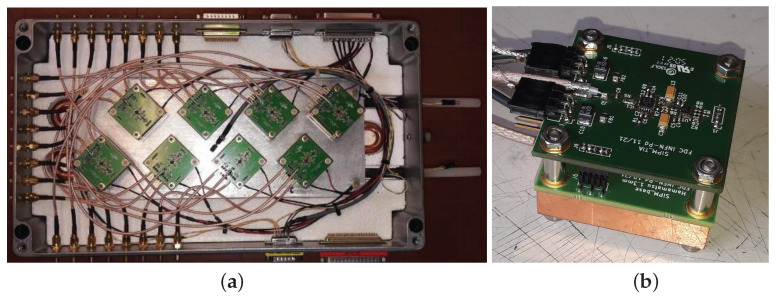
(**a**) the dark box with 8 SiPM modules mounted over a cooling plate. (**b**) the SiPM module.

The setup used to characterize SiPMs is shown in Figure 1a.

Eight SiPM modules (Figure 1b) are placed in the metallic box (see Figure 1a). The metallic box has two insulating layers (on its top and bottom); in between of them it stands a cold plate connected with a chiller (minichiller Huber 600 OLÈ [12]). The chiller can bring the cooling liquid to a temperature of −20 °C. In the metallic box several temperature and humidity probes provide a good environmental monitoring of the system.

**Figure 2:**
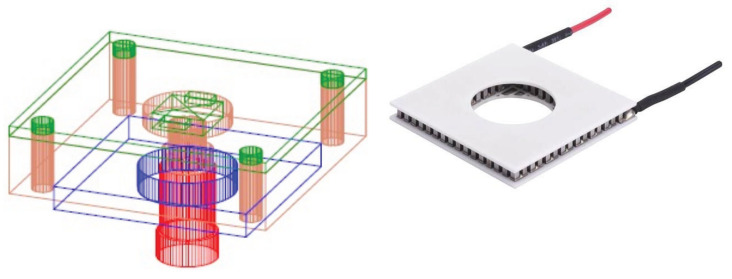
On the left the scheme illustrating the positioning of the Peltier cell with respect to the copper block; the red cone in the center is the quartz fiber head illuminating the SiPM. On the right the Peltier cell with a central hole to allow the laser illumination.

A SiPM module is composed of a Peltier cell (see Figure 2 on the right), whose hot face is in contact with the cold plate and the cold face with a copper block.

A board hosting the SiPM is placed on top of the copper block; the copper block provides a uniform cooling surface to keep the whole SiPM board at the given temperature. On this board two sensors are placed in order to control the SiPM temperature. The board allows a precise positioning with respect to the quartz fiber illuminating the SiPM (see Figure 2 on the left).

The amplification board is placed on top of the SiPM board (see Figure 1b). The signal amplification is provided by a custom low-noise high-bandwidth transimpedance amplifier build with a Texas Instruments THS4303 [13] operational amplifier.

The module is designed in such a way that only the SiPM boards are replaced when different SiPM sets are studied. For all these measurements we used the same amplifiers in the same box position.

The combined cooling action of the cold plate and Peltier allows bringing SiPM temperatures down to −40 °C and keeping them at the desired value within 1mK. The box is flown with nitrogen in order to reduce humidity inside it.

A pulsed laser diode PiLAS model PiL040X [14], with a wavelength of 405 nm and pulse width less than 17 ps, is used to illuminate the photodetectors. In order to get the best timing performances the laser must work with large intensities, but we are interested in studying the device response to a single-photon. To reach the desired low intensity the laser beam is first attenuated with a mechanical attenuator, and then split into 8 quartz fibers. Each optical fiber is inserted into a SiPM module to illuminate it. The beam intensity on each SiPM is different because of the way the beam is splitted.

A control system, based on a Xilinx Zynq UltraScale+ processor [15], is responsible for controlling SiPM bias voltage and temperatures, providing the power supply to the electronics, driving the Peltiers and monitoring temperatures, humidity and the chiller.

**Figure 3:**
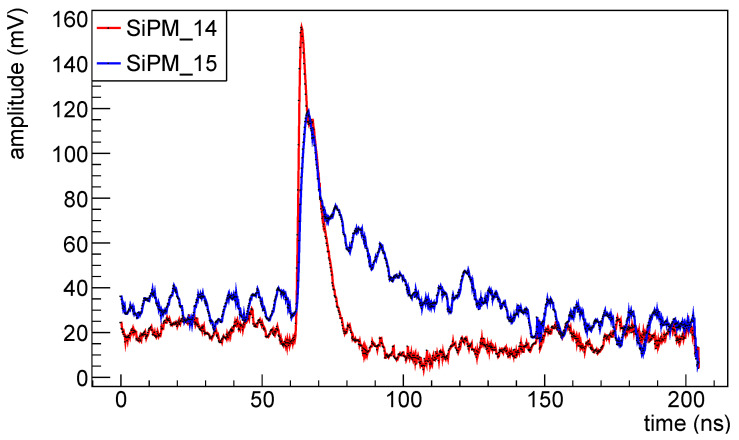
Waveforms for two different SiPMs before irradiation: in red SiPM #14 (FBK $1\times 1\;{\mathrm{mm}}^{2}$) and in blue #15 (Hamamatsu $3\times 3\;{\mathrm{mm}}^{2}$).

Signals are acquired with a V1742 CAEN VME digitizer [16] that can operate at 5GS/s; waveforms were recorded and processed offline (except for the first data taking for H1 set, before first irradiation, for which only max amplitudes and arrival times were recorded). In Figure 3 two typical waveforms, one for a $1\times 1\;{\mathrm{mm}}^{2}$ FBK device and one for a $3\times 3\;{\mathrm{mm}}^{2}$ Hamamatsu one are shown.

Each device set has gone through different irradiation and annealing campaigns.

SiPM boards have been irradiated at the LNL INFN laboratory by using the 0° line of the CN accelerator. A deuterium beam of 4 MeV with a current of up to 100 nA was sent on a beryllium target 50 μm thick with a 72-degree angle. SiPMs were not powered while being irradiated.

**Figure 4:**
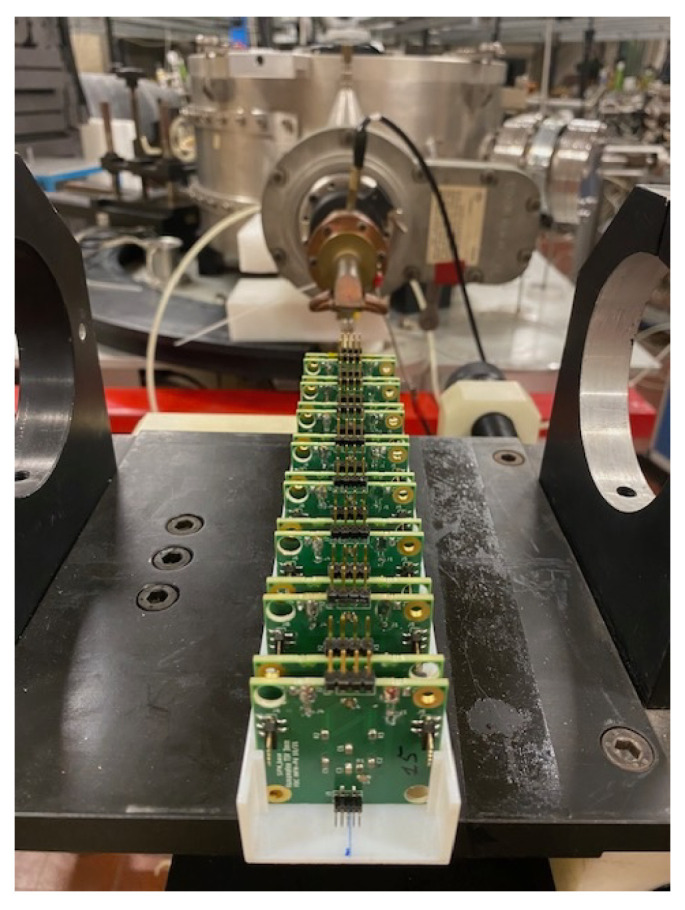
SiPMS board irradiated inside the CN beam line.

Figure 4 shows 16 SiPM boards placed downstream of the target. Different radiation levels can be obtained by acting on the distance and exposure time. In Table 3, Table 4 and Table 5 the accumulated equivalent number of neutrons in each campaign is reported respectively for device set H1, FBK1 and MIX1. Device set H2 was irradiated just once, in April 2024; for this sample all SiPMs got the same dose of $1.0\times 10^{10}$ neq/cm^2^.

SiPMs were annealed after irradiations. SiPM set H1 was annealed at 150 °C for 8 weeks after first irradiation and at 80 °C for two separated periods of 1 day and 7 days after second irradiation. Set FBK1 was annealed at 150 °C for 8 weeks after first irradiation and at 80 °C for 1 day after second irradiation. At the moment the other two sets have not been annealed yet, they will be in the next future.

**Figure 5:**
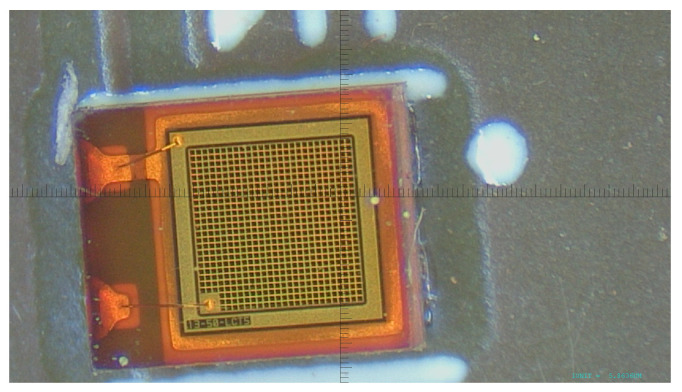
Microscope picture of SiPM #0.

The SiPMs of set H1 were damaged during the first annealing campaign: the window material is made of epoxy resin and after annealing it was no longer transparent to the laser photons, but the SiPM is working, so dark count rate measurements are still possible. A microscope image shows the input surface of SiPM # 0 (Figure 5); after annealing the surface shows a brownish color.

The devices have been studied before irradiation and after each step: non-irradiated, irradiated, annealed, irradiated2, annealed2 and annealed3.

For each device set, several measurements at different temperatures and different bias voltages were performed (or will be, work is still in progress): IV-curves, dark count rate, amplitude and timing. The IV-curves were measured with a Keithley 6487 pico-ammeter [17] for each step.

Dark count rate was measured acquiring the signals with the CAEN digitizer with random triggers.

Amplitude spectra were acquired illuminating the SiPMs with the pulsed laser; from these data we got several information among which the separation between the photon peaks and the time resolution. The pulsed laser provides the trigger signal to the digitizer, with this information we get the precise arrival time of the photons on the SiPM; with large signals we can reach laser resolutions of the order of 20 ps.

The laser and DCR data were taken varying the bias voltage on an interval of at least 1.6 V with steps of 0.1 V. In general the bias voltage interval was the same for all data sets, but, for set FBK1 we changed drastically after the first irradiation as the previously used interval was providing signals too small to be analyzed.

## 3. Software, Analysis Tools and Methods

Except for set H1, for which before irradiation the amplitude and time were extracted online during data taking, for all others and for set H1 after first irradiation we stored the waveforms for offline processing.

**Figure 6:**
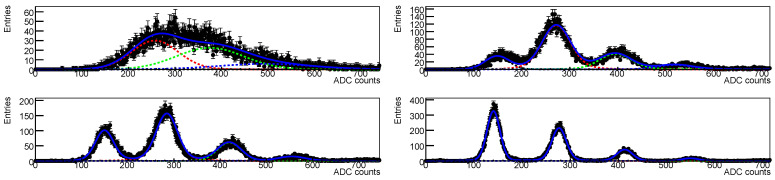
Photon spectra for SiPM #2, after first irradiation at $1.01\times 10^{11}$ neq/cm^2^, at different temperatures. From top to bottom spectra taken at temperatures of 10 °C, −10 °C, −20 °C and −35 °C. The spectra are taken at the same overvoltage (2 V). The colored curves correspond to the different photon peak contributions to the poissonian.

With the amplitude we get a spectrum as one of those shown in Figure 6; in these plot (except for the top left one) we see a sequence of gaussian distributions, each gaussian peak is given by the detection of *n* photons, and the relative population should be driven by the Poisson statistics; in this case we have a distribution with signals up to four visible photons. These spectra are obtained by selecting the signals whose timing is in a window of few ns around the expected signal time.

These amplitude spectra are fitted, using RooFit [18,19,20], with a convolution of a poissonian distribution, that describes the photon emission statistics, and gaussian distributions the describe the signal corresponding to *n* photons; this distribution is affected by two processes: prompt and delayed crosstalk.

Prompt crosstalk consists in an avalanche generated in a nearby pixel by a photon generated in the primary avalanche; the timing of the two avalanches is essentially the same. The effect of the prompt crosstalk in the poisson distribution is to reduce the population of the peak corresponding to the number *n* of avalanches generated by photons coming from the laser beam and increasing the population of the nearby distribution, corresponding to the n+1 term of the poissonian. The prompt crosstalk is fitted by setting, for each photon, a probability factor *f* to generate an extra signal: the poissonian probability for n−photons peak is reduced by a factor n×f and the (n+1)−photons peak probability is increased by the same quantity; this is done recursively starting from the single-photon probability.

With delayed crosstalk, instead, a single-photon signal is generated by an electron drifting in a nearby cell; this signal overlaps with the true signal with a small delay, due to the drift time, that causes its contribution to be summed to the original signal as a single-photon signal on the descending tail not fully contributing to the leading edge. As a consequence the signal is a little smaller than what it should be if the crosstalk photon had been emitted at the same time of the original signal, and it shifts the time measurement by a small amount related to the fact that the constant fraction threshold is lowered w.r.t. what it should be if the avalanche was in time.

On the single-photon signal there’s no crosstalk contribution.

The photon peaks are fitted with two gaussian describing the photon peak amplitude, one gaussian corresponds to the prompt signal, the second one gives the contribution of the out of time delayed crosstalk. The amplitude difference between two contiguous peaks from the prompt signal is fixed, the component from the delayed crosstalk is let to vary around the position of the main gaussian.

The main gaussian width of each photon peak is described by the sum in quadrature of a constant term ($\sigma_{0}$, due to electronic fluctuation), equal for all the photon peaks, and of a term proportional to the photon number ($\sigma_{1}$, due to intrinsic fluctuation of every involved microcell).

From the acquired signal (see Figure 3) we extract the max amplitude and, from this, the timing of the signal; the timing is extracted with the constant fraction discriminator method, with a fraction set to 50% and is referenced to the trigger time, extracted from the digitizer data by using the constant fraction discrimination technique too.

The analysis of these data is reported in the following sections.

## 4. Results

### 4.1. IV Characterizations

The reverse IV-curves at temperatures from 20 °C to −40 °C have been taken for all steps; unfortunately for device set H1 the data before irradiation were lost, but the curves for all the devices were almost superimposing, with a small shift due to the slightly different breakdown voltage for each SiPM.

**Figure 7:**
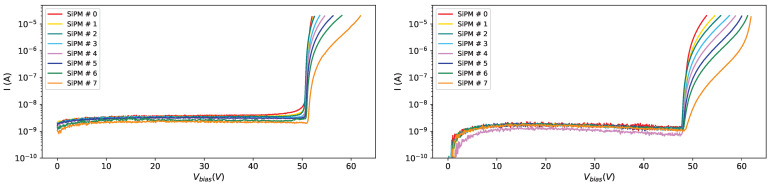
IV-curves for SiPMs of set H1 after first irradiation campaign. Curves for all SiPMs, at 10 °C on the (**left**) and at −40 °C on the (**right**).

From Figure 7 we can see the effect of the radiation dose on the current at different temperatures (10 °C on the left and −40 °C on the right): the curve becomes steeper and steeper as the radiation dose increases, and this effect is visible at different temperatures, even if, looking at the right plot in Figure 7 we can see that at low temperatures (−40 °C) the IV-curves for different irradiations tend to be more separated than at higher temperatures.

**Figure 8:**
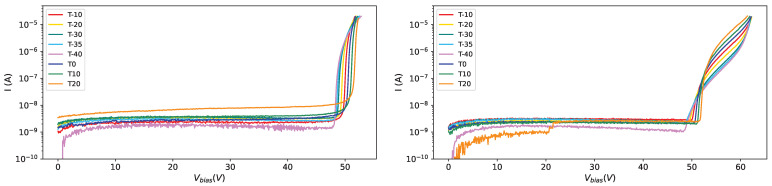
IV-curves for SiPMs of set H1 after first irradiation campaign. Curves for all temperatures, for SiPM #0 on the (**left**) and for SiPM #7 on the (**right**).

In Figure 8 the IV-curves for SiPM # 0 (the most irradiated one) on the left and for SiPM # 7 (the less irradiated one) on the right are shown at various temperatures after the first irradiation campaign.

**Figure 9:**
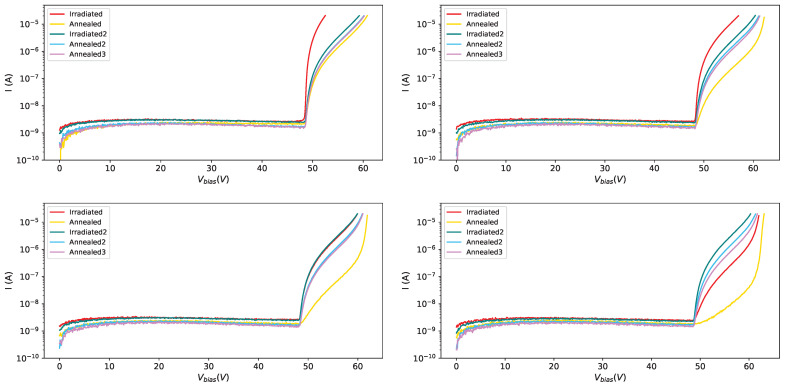
IV-curves at −35 °C: in red after first irradiation, in yellow after first annealing, in teal after the second irradiation campaign, in light blue after second annealing and in magenta after third annealing; SiPM # 0 on (**top left**), SiPM # 3 on (**top right**), SiPM # 5 on (**bottom left**), SiPM # 7 on (**bottom right**).

In Figure 9 the effect of irradiation and annealing is shown for four different SiPMs of set H1. It is interesting to observe SiPM #5: it received the same amount of radiation in the two campaigns, with the annealing in between; the IV-curves, after irradiations, almost overlap; it must be observed also that the first annealing (150 °C) had a strong effect on the IV-curve while the other two (80 °C for one day and one week respectively) had the same effect.

**Figure 10:**
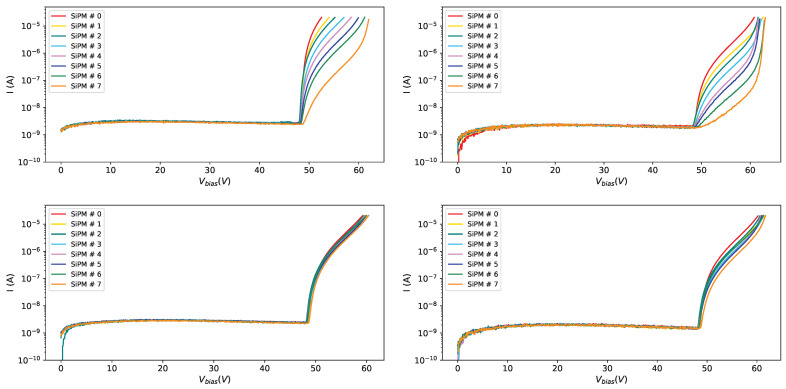
IV-curves at −35 °C for set H1: after first irradiation (**top left**), after first annealing (**top right**), after second irradiation (**bottom left**) and after third annealing (**bottom right**).

It is interesting to compare the IV-curves along the steps followed for the H1 set (see Figure 10). The different radiation dose affects strongly the IV-curves (Figure 10 top left), the first (strong) annealing (Figure 10 top right) has an effect on the current, lowering it, but makes the curves even more different among each other; after the second irradiation campaign (Figure 10 bottom left), were all the devices were exposed to the same radiation dose, all the IV-curves are very similar, almost superimposing, while after the third annealing (Figure 10 bottom right) we see that the IV-curves are not superimposing as before annealing, showing that there’s a memory effect on previous irradiations.

Another observation, already reported by others ([5] for example) is the fact that most of the recovery is obtained in the first 24 h.

**Figure 11:**
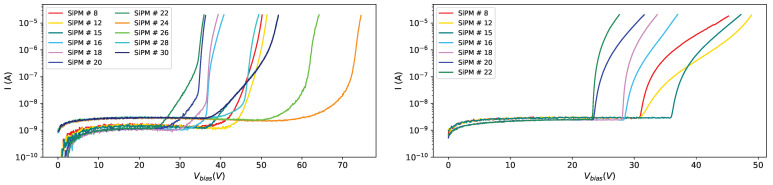
IV-curves for SiPMs from different producers at temperature of 20 °C before irradiation (**left**) and after first irradiation at $1\times 10^{10}$ neq/cm^2^ (**right**).

Figure 11 shows the reverse IV-curves for SiPMs of different producers at temperature of 20 °C before and after irradiation. To make the comparison meaningful, SiPMs with equal irradiation levels ($1\times 10^{10}$ neq/cm^2^) have been considered.

### 4.2. Photon Spectra

From the acquired signal (see Figure 3) we extract the max amplitude and, from this, the timing of the signal.

**Figure 12:**
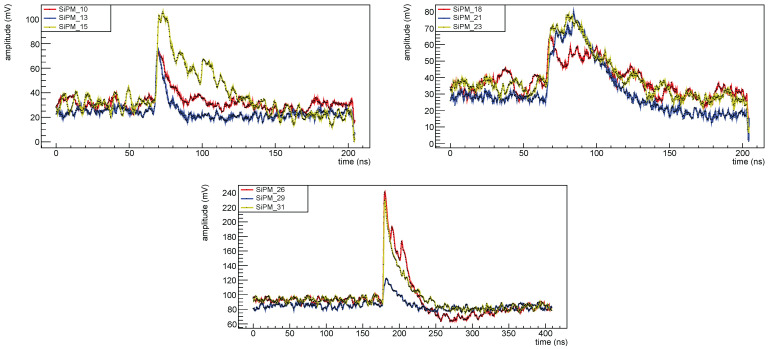
Waveforms before irradiation at 10 °C. Set FBK1 (**top left**): red for SiPM #10, blue for SiPM #13, yellow for SiPM #15. Set MIX1 (**top right**): red for SiPM #18, blue for SiPM #21, yellow for SiPM #23. Set H2 (**bottom**): red for SiPM #26, blue for SiPM #29, yellow for SiPM #31.

The signal shape is quite different for different devices. In Figure 12 top left, the waveforms for SiPM #10 ($3\times 3\;{\mathrm{mm}}^{2}$ FBK) in red, SiPM #13 ($1\times 1\;{\mathrm{mm}}^{2}$ FBK) in blue, and SiPM #15 ($3\times 3\;{\mathrm{mm}}^{2}$ Hamamatsu) in yellow are shown: from the waveform it appears that $3\times 3\;{\mathrm{mm}}^{2}$ devices are much noisier than $1\times 1\;{\mathrm{mm}}^{2}$ (this is expected because the noise is proportional to the capacity of the SiPM, which in turn is proportional to its size) and this makes the information extraction more difficult; in fact we were not able to extract any information from $3\times 3\;{\mathrm{mm}}^{2}$ FBK devices.

In Figure 12 top right, waveforms from devices from set MIX1 are shown; also for the $3\times 3\;{\mathrm{mm}}^{2}$ devices from this set it was not possible to extract any information from the amplitude spectra, while for the $1\times 1\;{\mathrm{mm}}^{2}$ OnSemi devices some information was extracted but for these SiPMs the time resolution is quite poor, not at the level of the experiment requirements.

**Figure 13:**
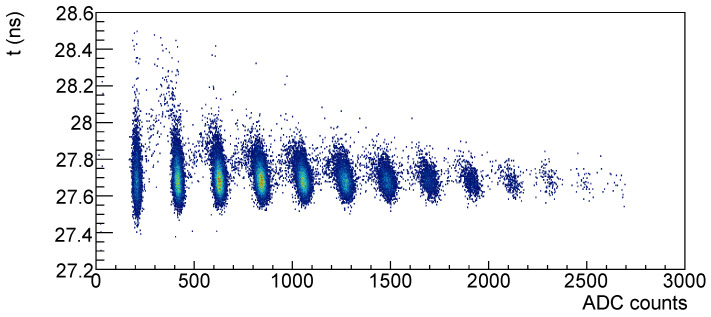
Time vs. amplitude for Hamamatsu SiPM #7 before irradiation.

In Figure 13 the time vs. amplitude correlation is shown; it can be noticed that as the number of signal photons increases the time resolution improves, but also that there seems to be a rotation of the distribution as the number of signal photon increases.

**Figure 14:**
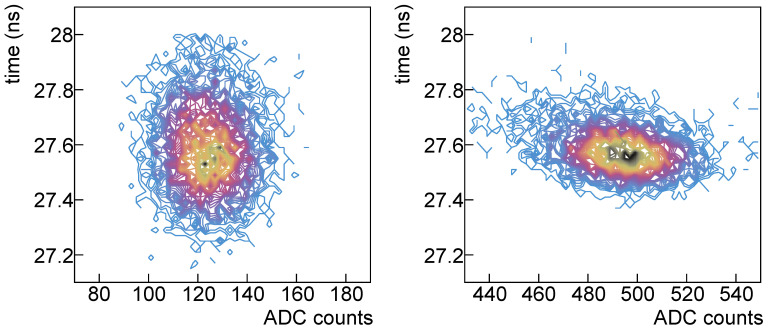
Time vs. amplitude for single-photon signal (**left**) and for four-photons signal (**right**).

Looking at Figure 14, where the single-photon and four-photons scatter plots are shown with the same scales, it is evident that the time resolution improves with the signal number of photons but there’s a distortion effect w.r.t. the plot on the left.

From the amplitude distribution, as explained in Section 3, we extract some information among which the *gain* (that is the separation between two nearby photon peaks), the peak widths, the prompt crosstalk probability.

### 4.3. Breakdown Voltage

**Figure 15:**
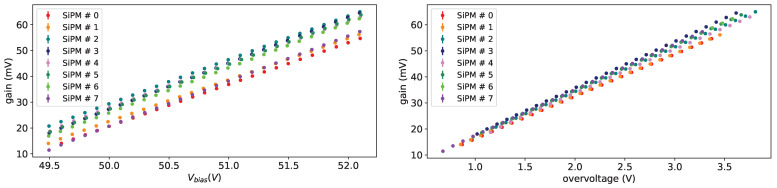
Gain vs. $V_{bias}$ (**left**) and vs. overvoltage (**right**) for all SiPMs of set H1 at T = −30 °C before irradiation.

Taking data at different bias voltages in given conditions allow us to extract the breakdown voltage ($V_{break}$), defined as the bias voltage where the gain is equal to 0, fitting the gain versus the bias voltage with a straight line. As an example on from left plot in Figure 15 we see a nice linearity of the gain w.r.t the bias voltage.

Based on the breakdown voltage, the overvoltage is defined as the difference $V_{bias}-V_{break}$.

In the left plot of Figure 15 the gain vs. bias voltage is shown for all SiPMs of set H1 before irradiation at a temperature of −30 °C. In the right plot of Figure 15 the same gain as a function of overvoltage is shown; from this plot we can see a small difference in the slopes.

**Figure 16:**
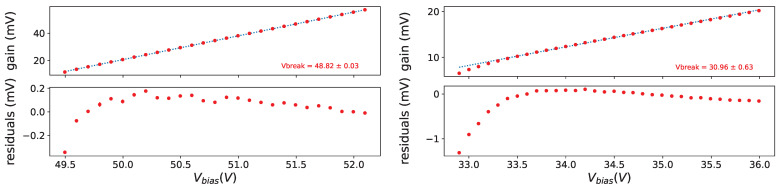
Gain vs. $V_{bias}$ for SiPM #7 (**left**) and #11 (**right**) at T = −30 °C before irradiation on (**top**), residuals of linear fit on (**bottom**).

In Figure 16 the gain as a function of the bias voltage is shown for SiPMs #7 and #11 before irradiation at a temperature of −30 °C on top. A good linearity can be seen, except at small bias voltages; for the Hamamatsu device the deviation is explained by the fact that photon peaks at this low voltages overlap quite a bit and the fitting procedure tends to find smaller separations between the peaks.

For the other device (FBK $1\times 1\;{\mathrm{mm}}^{2}$) the explanation is different: the separation between peaks is good enough and the smaller separation seems to be related to the avalanche generation mechanism.

For the extraction of $V_{break}$ the points at lower $V_{bias}$ are excluded where the photon peaks are overlapping too much, and for FBK devices, even if the peaks are well separated, we exclude the points at low $V_{bias}$ with large residuals.

With this data we can extract the breakdown voltages (as previously defined) and gain variations with bias voltage; we performed this analyzed at different temperatures (see, for example, Table 6 and Table 7).

**Figure 17:**
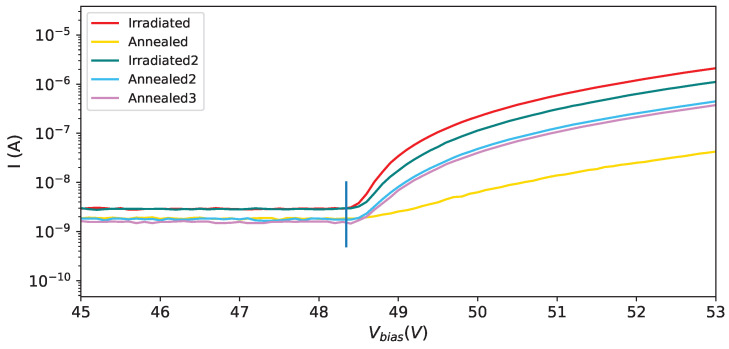
Expanded view of IV-curve for SiPM #4 at −30 °C: the teal vertical line shows the breakdown voltage obtained from the gain vs. bias voltage fit.

In Figure 17 an expanded view of the IV-curves for SiPM #7 at various stages is shown with superimposed the breakdown voltage obtained at this temperature by fitting the gain as a function of the bias voltage.

#### 4.3.1. Set H1

The breakdown voltages have been measured by fitting the gain vs. $V_{bias}$ distributions and extrapolating the intercepts with the *x*-axis.

This is an important parameter as, from it, we can determine the overvoltage, that is the parameter that, within the same device family, defines the working point together with the temperature.

Breakdown voltages have been extracted for all SiPMs at different temperatures at each step. In Table 6 their values, with their errors, are reported for set H1 before irradiation. We can see that $V_{break}$ for these 8 devices stays in an interval of 0.5 V, at any temperature.

As expected, there is no significant change in $V_{\mathrm{break}}$ as fluence increases but there is a significant increase in error due to the increased error in gain estimation.

**Figure 18:**
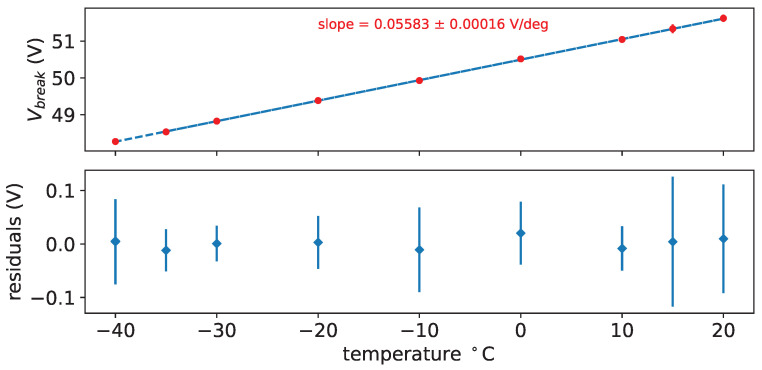
Breakdown voltage (V) vs. temperature for SiPM # 7 before irradiation. In the bottom plot residuals of the fit are shown.

We studied also the variation of the breakdown voltage with temperature; in Figure 18 the breakdown voltage as a function of temperature is shown for SiPM #7 before irradiation, while in Table 8 the thermal coefficient derived from the fits, with their errors, is reported.

**Figure 19:**
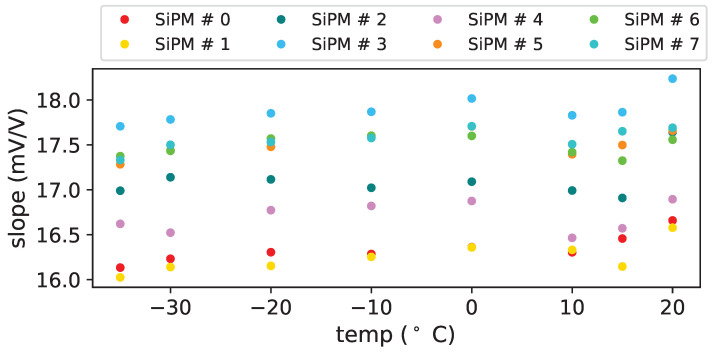
Slope vs. temperature for SiPMs of set H1.

From the fits we extract also the gain variation over bias voltage, these values for non irradiated devices are reported in Table 7. From the table we can see that there’s a small dependency on the temperature (the relative variation is of the order of $10^{-4}{/}^{\circ }C$ (see Figure 19) and a larger variation among devices.

#### 4.3.2. Set FBK1

In Table 9 the breakdown voltages at different temperatures are listed for $1\times 1\;{\mathrm{mm}}^{2}$ FBK SiPMs and for the $3\times 3\;{\mathrm{mm}}^{2}$ Hamamatsu of set FBK1, while in Table 10 the gain variation with bias voltage at different temperatures is reported.

For the $3\times 3\;{\mathrm{mm}}^{2}$ FBK devices it was not possible to extract any meaningful amplitude spectra with clear photon peak separation, hence it was not possible to study their behavior as for the other devices.

In Table 9 for SiPM #11 $V_{break}$ is reported only for some temperatures because this device shows some peculiar behavior.

**Figure 20:**
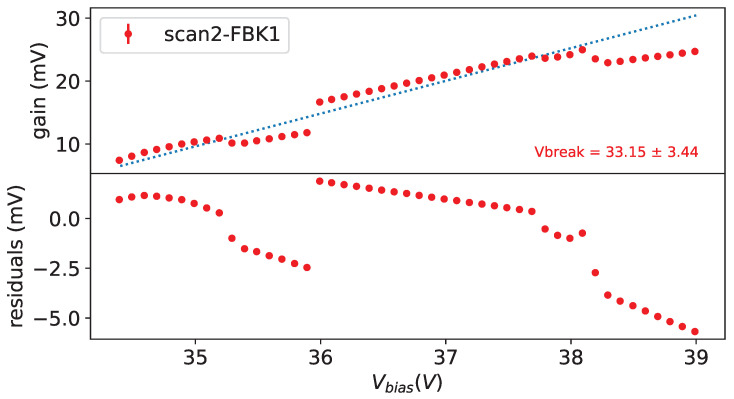
Gain vs. $V_{\mathrm{bias}}$ for SiPM FBK #11 before irradiation at T = 10 °C. On the top plot the blue dots show the interpolated line.

Figure 20 shows the gain vs. $V_{bias}$ at a temperature of 10 °C: clear discontinuities are visible at different locations; same effect is present in scans at different temperatures, but these jumps occur at different overvoltages, so there’s no simple explanation for them.

A detailed inspection of the data didn’t show any evident problem in the data itself. No other device showed such a behavior.

In Table 11 the breakdown voltages at different temperatures are listed for set FBK1. For the $3\times 3\;{\mathrm{mm}}^{2}$ FBK devices it was not possible to extract any meaningful amplitude spectra with clear photon peak separation, as before irradiation. For SiPM #15 at 20 °C it was not possible to extract the amplitude spectra, while we could do it at lower temperatures. After irradiation SiPM #11 showed discontinuities only at a temperature of 0 °C, but it must be noted that these bias voltage scans had a smaller range w.r.t. the scans before irradiations: only 1.6 V. Also the bias scan covered higher bias voltages, at least for FBK devices.

**Figure 21:**
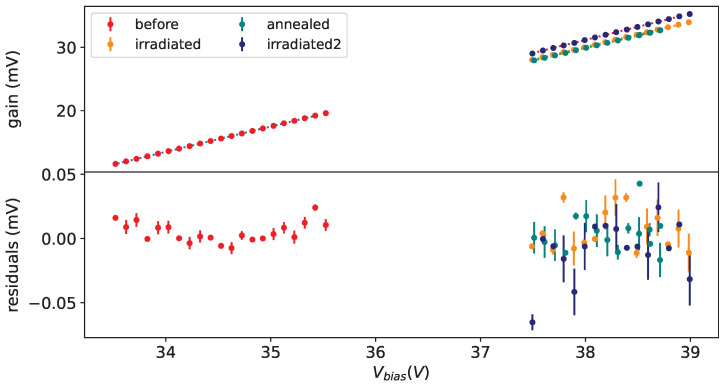
Gain vs. $V_{\mathrm{bias}}$ for SiPM FBK #12 at T = −35 °C, before first irradiation (red), after first irradiation (orange), after annealing (teal) and after second irradiation (blue).

Figure 21 shows the gain as a function of bias voltage for SiPM #12 before irradiation, after first irradiation, after first annealing and after second irradiation at the temperature of −35 °C. For this SiPM both irradiations were at $1\times 10^{10}$ neq/cm^2^. The results of the fits are reported in Table 9 and Table 11, Table 12 and Table 13.

In Table 14 the fitted breakdown voltages, with their error, are listed for some SiPMs before and after irradiation at various temperatures.

For the FBK $1\times 1\;{\mathrm{mm}}^{2}$ devices, unlike H1 set, breakdown voltages are not the same before and after irradiation: comparing Table 9 with Table 11 we can see that $V_{break}$ values before and after first irradiation are not compatible within the measurement error.

Comparing Table 9, Table 11 and Table 12 we can see that there is no significant change in $V_{\mathrm{break}}$ for different fluences, after annealing, after re-irradiation.

#### 4.3.3. Set H2

In Table 15 the breakdown voltages for SiPM set H2 before irradiation are listed. These eight devices have in common the detection area ($3\times 3\;{\mathrm{mm}}^{2}$), but have different pixel size and are of different type (see Table 1). We measure different breakdown voltages for the two series (13,360 for the first four and 14,160 for the last four) compatible with producer’s datasheets.

We observe that the small pixel devices (for example SiPMs #24 and #25 (with 25 μm cell size), #28 and #29 (with 15 μm cell size) in set H2) have a smaller gain and a smaller gain variation with the overvoltage (see Table 16). This is expected, but it also means that to have a good signal to noise ratio the devices must be operated at very large overvoltages, as is the case for the FBK $1\times 1\;{\mathrm{mm}}^{2}$ ones; in fact from our measurements we can say that one should work at least with an overvoltage of 9 V with them.

### 4.4. Signal Analysis

The effect of irradiation is to introduce a noise that affects both the baseline and the signal, making difficult to extract the information.

**Figure 22:**
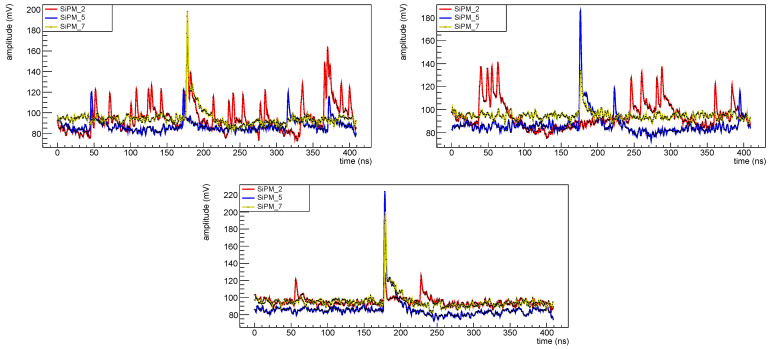
Waveforms for SiPM # 2 (red), 5 (blue) and 7 (yellow), of set H1, after first irradiation campaign: on the (**top left**) at 10 °C, on the (**top right**) at −10 °C and on the (**bottom**) at −30 °C. These waveforms were taken all at an overvoltage of 2 V.

In Figure 22 the waveform for SiPMs #2 ($1.01\times 10^{11}$ neq/cm^2^, red), #5 ($1.02\times 10^{10}$ neq/cm^2^, blue) and #7 ($1.03\times 10^{9}$ neq/cm^2^, yellow) taken at different temperatures are shown superimposed: it is clearly visible the noise level increasing with radiation dose and decreasing with decreasing the temperature. As a consequence for the most irradiated devices it is possible to extract the amplitude spectra only at low temperatures; this can be clearly seen in Figure 6.

Spectra taken for SiPM #2 at various temperatures but at the same overvoltage after first irradiation are shown in Figure 6; we see that already at −10 °C (second plot from top) it is possible to separate the photon peaks, even if the noise makes the peaks to overlap. Lowering the temperature the peaks are better separated due to the decrease in the noise.

The data was taken with the same laser intensities; the difference in the relative population of the photon peaks, especially that of the single-photon peak, can be explained by a problem in the peak finder. The peak finder looks for the maximum amplitude signal but with a noisy environment, where the noise is given by signal corresponding to one or more photons; in fact the algorithm may find the first peak at a different time w.r.t. laser beam, this will depopulate the single-photon peak associated with the laser pulse. To take into account this effect in the fit function a parameter taking into account the inefficiency on the single photon was added.

As it was shown, working at low temperatures in general improves the signal detection, but we observed also some drawback: in fact, at least from the point of view of the amplitude, for some devices at low temperatures the signal is degraded.

**Figure 23:**
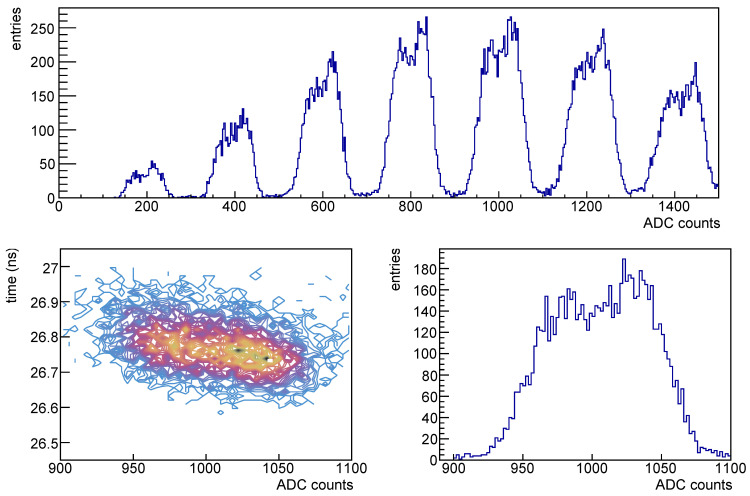
SiPM #4 at 0 °C: on (**top**) amplitude spectrum, on (**bottom**) plots relative to the five photon signal: on the (**left**) the time vs. amplitude and on the (**right**) the amplitude.

One example, at 0 °C, can be seen in Figure 23, where the amplitude spectrum is shown in the top plot, for the five photon signal the time vs. amplitude on the bottom left and the amplitude on the bottom right one. The photon peaks in the amplitude spectra (top plot of Figure 23) are wider than in typical distributions and they show the contribution from two gaussian distributions slightly displaced, this is more evident in the bottom right plot of the same figure, where the amplitude for the five photons signal is shown.

In the five photon amplitude plot of Figure 23 (bottom right) it is visible a splitting of the amplitude distribution, as if the avalanche, and hence the internal amplification, was not stable but jumped between two values. We exclude that this effect was related to the amplifier.

We checked the signal evolution during data taking and this effect is constant along the whole data sample. Even in the time vs. amplitude plot Figure 23 (bottom left) it is possible to see the amplitude splitting, while on the timing there’s not such an evident effect, and on the time resolution it seems not to be important. Further investigations on this effect are required.

**Figure 24:**
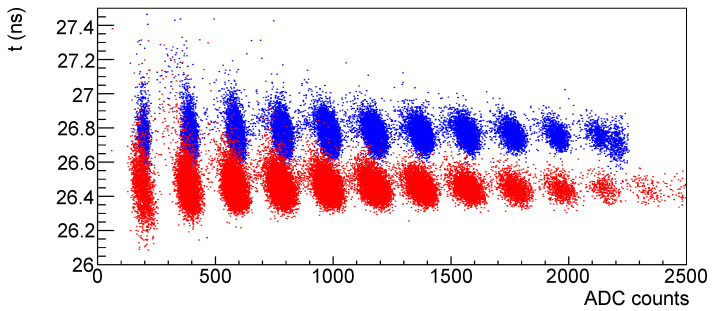
Time vs. amplitude distributions for SiPM # 4 at 20 °C in blue and at −30 °C in red. Both distributions are taken at the same overvoltage.

This effect can also be seen comparing the time vs. amplitude plots at two different temperatures (Figure 24 for SiPM #4): in blue data taken at 20 °C while in red data taken at −30 °C. We can see that, while the time width of the peaks is essentially unchanged, the amplitude width is much larger at the lower temperature.

**Figure 25:**
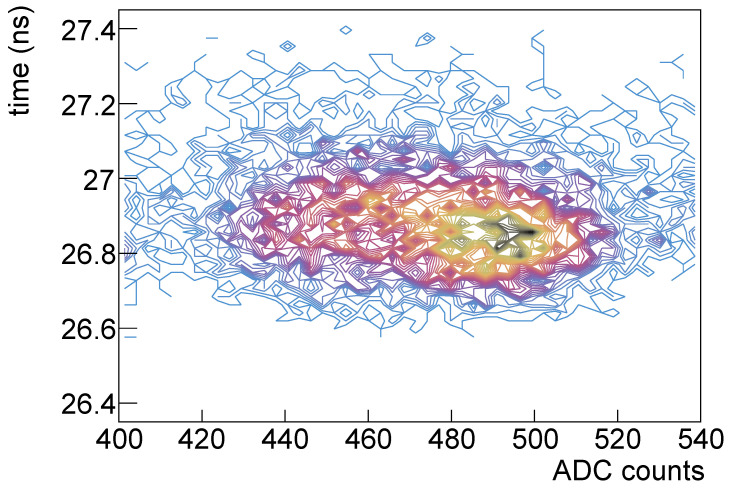
SiPM #26 at 0 °C: time (ns) vs. amplitude (ADC).

This effect was seen also in other devices, more or less evident and at different temperatures; in Figure 25 the time vs. amplitude plot for SiPM #26 ($3\times 3\;{\mathrm{mm}}^{2}$ Hamamatsu) at a temperature of 0 °C is shown for the four-photons signal.

**Figure 26:**
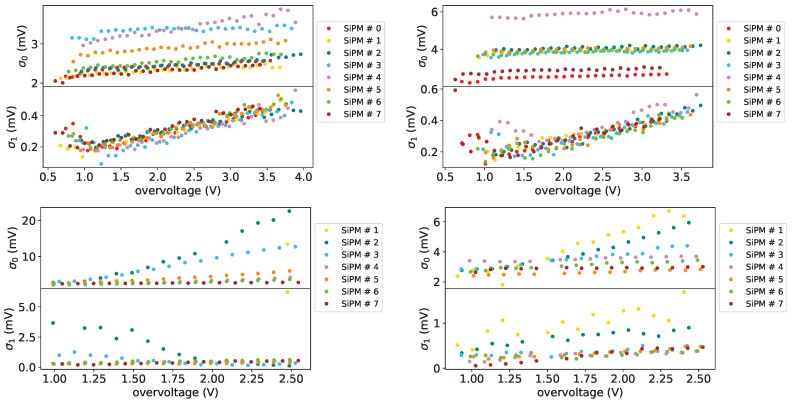
Photon peak width parameters for device set H1, top plots before irradiation, at 10 °C on the (**left**) and −20 °C on the (**right**); bottom plots after first irradiation, at 10 °C on the (**left**) and −20 °C on the (**right**). On (**top**) panel the constant term $\sigma_{0}$, on (**bottom**) panel the variable term $\sigma_{1}$.

In top plots of Figure 26 the width (in mV) of the photon peaks is shown for two temperatures (10 °C on the left and −20 °C on the right) for all the SiPMs of set H1 before irradiation. We can see that the variable part $\sigma_{1}$ (in the bottom panel of the plots) is quite small and has a *relatively strong* dependency on the overvoltage, while the constant term $\sigma_{0}$ (in the top panel of the plots) is less affected by the overvoltage.

Another interesting result, that is consistent with what reported earlier, is the fact that the constant term $\sigma_{0}$ for H1 set devices tend to increase when lowering the temperature, while the variable one seems not to be affected by the temperature. The behavior of the $\sigma_{0}$ and $\sigma_{1}$ parameters at low overvoltages can be explained by the fact that in this region the photon peaks are partially superimposing and in this situation the fit has some problems in discriminating between the two contributions.

**Figure 27:**
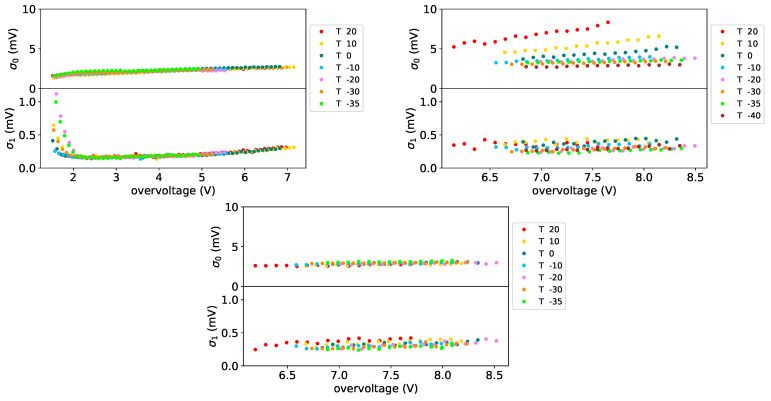
Photon peak widths parameters (in mV) for SiPM #12 (set FBK1) as a function of the overvoltage (in V) for different temperatures: on the (**top left**) before irradiation, on the (**top right**) after first irradiation on the (**bottom**) after first annealing. On top panel the constant term, on bottom panel the variable term.

In Figure 27 the peak width parameter as a function of the overvoltage is shown for SiPM #12 (SiPM set FBK1) for different temperatures, before irradiation on the top left, after first irradiation on the top tight and after first annealing on the bottom.

Comparing the plots of Figure 27 with those of Figure 26 we can notice that for these devices there’s a little dependence on the overvoltage for these two parameters.

From FBK1 set we see that the FBK devices seem to be more uniform with temperature than the H1 devices before irradiation and after first annealing; after first irradiation the peak width depends on the temperature, this can be explained with the effect of the noise on the signal.

From the fit we extract the average number of photons, assuming a poissonian distribution. This parameter, in our setup, is not completely stable, as it depends on the input illumination of the beam splitter and disconnecting/reconnecting the fibers affects the distribution of the photons in the fibers, but, in general, is stable during a data acquisition session; we can then measure the variation of this parameter with the bias voltage, as the acquisition session covers the bias scan in a reasonable time interval.

**Figure 28:**
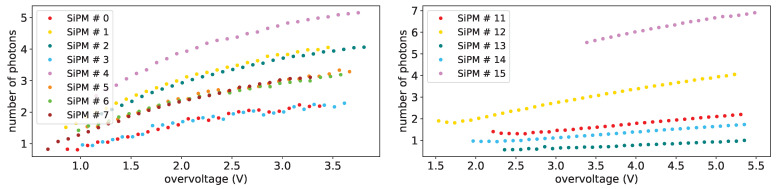
Average number of photons vs. overvoltage at T = −30 °C before irradiation for H1 set on lef and set FBK1 on (**right**).

In Figure 28 the average number of photons from the fit as a function of the overvoltage is shown for all the SiPMs of set H1 (on left plot) and for FBK1 ones (on right plot) at a temperature of −30 °C before irradiation. What we can note is the fact that it increases with overvoltage and the general trend is the same for all the SiPMs. This increase can be explained by an improvement in the detection efficiency of the devices. For $1\times 1\;{\mathrm{mm}}^{2}$ FBK devices (SiPM #11-#14 on right plot of Figure 28) we can see a more linear behavior with overvoltage w.r.t. H1 ones. In this figure SiPM #15 is a $3\times 3\;{\mathrm{mm}}^{2}$ Hamamatsu device. As explained before the data at small overvoltages are affected by the superposition of the photon peaks that makes the fit not completely reliable.

It must be stated that the stability of the laser was studied in the past for several months with a different apparatus and at constant SiPM conditions, we didn’t measure any variation in the average number of photons, what we observed was a slight dependency of the laser timing with the laser head temperature, measured in 45 ps/°C; in our data this has no significant effect as a typical datataking lasted of the order of 5 minutes.

**Figure 29:**
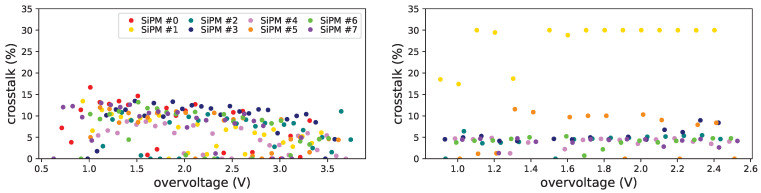
Prompt crosstalk vs. overvoltage for SiPMs of set H1 at −20 °C, before irradiation (**left**), after irradiation (**right**). Data could not be extracted after irradiation for SiPM #0 at this temperature. The legend is the same for the two plots. Zero values come from unreliable fits.

We studied also the prompt crosstalk (in the following called just crosstalk), as explained in Section 3. In Figure 29 the crosstalk before and after irradiation is shown for data taken at the temperature of −20 °C for set H1.

In H1 set the effect of irradiation is quite visible for the most irradiated SiPM (#1 in Figure 29), while for the less irradiated ones the difference is less evident. In this set the extraction of the crosstalk is not easy, but we can state that, for non irradiated devices, it is around 15% and is quite independent of the overvoltage, at least in the tested range. Due to the effect on the SiPM window we have no data on this item after annealing and further irradiations.

From this we can confidently exclude that the increase with overvoltage in the average photon number, shown in Figure 28 top, is related to an increase of the crosstalk.

The same information was extracted also for FBK1 set; for this set also the data after annealing are available.

**Figure 30:**
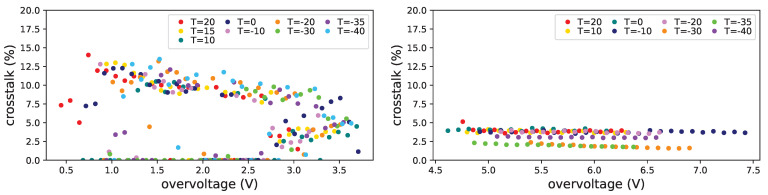
Prompt crosstalk as a function of overvoltage at different temperatures for Hamamatsu SiPM #6 (**left**) and for FBK #14 (**right**) before irradiation. Zero values come from unreliable fits.

The crosstalk is independent on the temperature both for Hamamatsu and FBK devices, as it is visible in Figure 30 (left and right respectively). From these data it appears that the prompt crosstalk extracted from these fits slowly decreasing with overvoltage.

**Figure 31:**
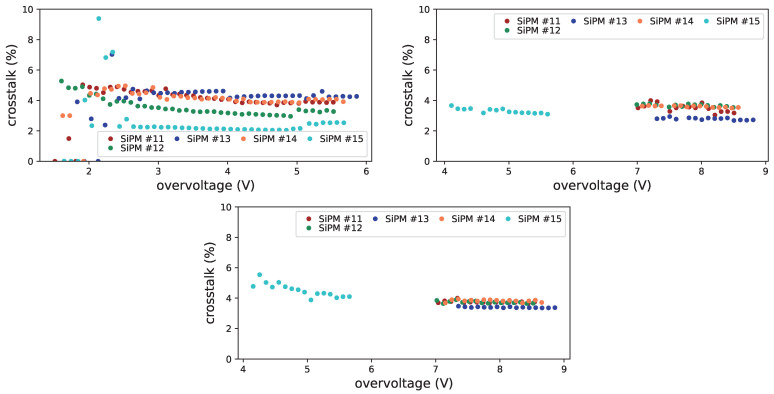
Prompt crosstalk vs. overvoltage for FBK1 SiPMs at −20 °C, before irradiation (**top left**), after first irradiation (**top right**) and after first annealing (**bottom**). Zero values come from unreliable fits.

In Figure 31 the crosstalk dependency on the overvoltage is shown before irradiation (top left), after first irradiation (top right) and after first annealing (bottom). From this set we were not able to get significant information on the $3\times 3\;{\mathrm{mm}}^{2}$ FBK SiPMs (#8, #9 and #10), but we could, even if with some difficulty, for Hamamatsu $3\times 3\;{\mathrm{mm}}^{2}$ SiPM (#15).

For these devices the crosstalk is smaller than for H1 (of the order of 4%) and seems not to be affected by the irradiation level at which they were subject. For the Hamamatsu device we can notice an increase after first irradiation and a further slight increase after first annealing, this has to be further investigated.

### 4.5. Time Measurements

The particle identification efficiency of the Time-Of-Propagation detector is related to the number of detected Cherenkov photons, the segmentation of photo-detection area and the time resolution. A combined time resolution between photodetector and readout electronics of about ∼100 ps is required, it’s important to check the impact of irradiation to the time resolution.

**Figure 32:**
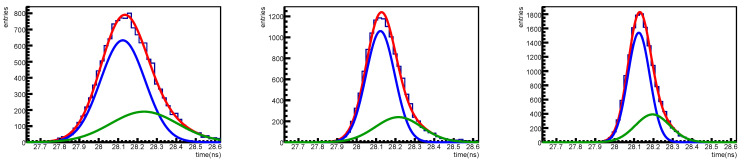
Time distributions for one photon (**left**) two photon (**center**) and more than two photon signals. The distribution is fitted with two gaussian distributions: in red the global fit, in blue and green the two gaussian contributions are shown. Distributions obtained for SiPM #4 at an overvoltage of 2 V and at 0 °C before irradiation.

The timing information is extracted as described in Section 3. Time distributions are obtained by selecting signals with amplitudes corresponding to single photon, two coincident photons and more than two photons; as an example the cut for the single-photon selection requires a signal between 0.5 and 1.5 the one photon amplitude reconstructed from the fit. In Figure 32 an example of time distributions is shown for SiPM #4 of set H1, the sigmas of the main (blue) gaussians is 115 ps for the single-photon signal, 74 ps for the two-photons one and 57 ps for more than two photons.

We will present results about time resolution only for sets H1 and FBK1, for the others the time sigma is not enough for our application being above 200 ps (OnSemi) or the analysis is still undergoing.

#### 4.5.1. Set H1

**Figure 33:**
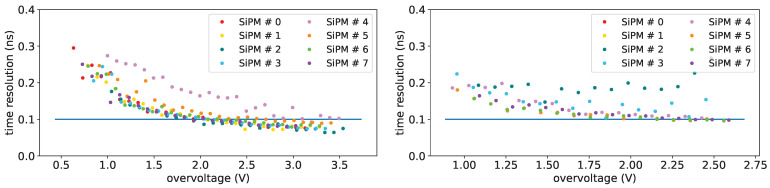
Single-photon time resolution vs. overvoltage for set H1 at a temperature of −10 °C before irradiation on (**left**) and after on (**right**). Horizontal line: reference for 100 ps.

Figure 33 shows the sigmas of the time distributions for the single-photon as a function of the overvoltage for SiPM set H1 before (on top) and after irradiation (on bottom) at a temperature of T = −10 °C. A sigma of about ∼100 ps can be obtained with an overvoltage of 2V on non irradiated devices, while for irradiated ones, at this temperature, it can be obtained for SiPM #5 (with an irradiation of $1\times 10^{10}$ neq/cm^2^).

For SiPM #4 (irradiation of $2.45\times 10^{10}$ neq/cm^2^) things are worst, but for this device we observed some degradation of its performances lowering the temperature.

For the two-photon signal we get a sigma better than 100 ps for overvoltages above 1 V both for non irradiated and irradiated devices at −10 °C.

**Figure 34:**
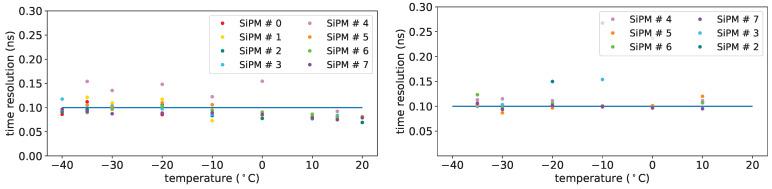
Single-photon time resolution vs. temperature for set H1 at an overvoltage of 2.5 V before on (**left**) and after on (**right**) first irradiation. Horizontal line: reference for 100 ps.

The single-photon sigma as a function of the temperature, for set H1, at an overvoltage of 2.5 V, is shown in Figure 34 before irradiation (on top) and after (on bottom).

It is interesting to observe that, for non irradiated devices there’s a slight worsening of time resolution as temperature is decreased, while for irradiated devices (at least for those for which it was possible to extract the time resolution) it is quite constant with the temperature.

For irradiated devices an impact on the extraction of the time resolution comes also from the peak finding algorithm: the inefficiency on the single-photon peak reduces the statistics and this brings to a degradation of the extracted resolution.

#### 4.5.2. Set FBK1

**Figure 35:**
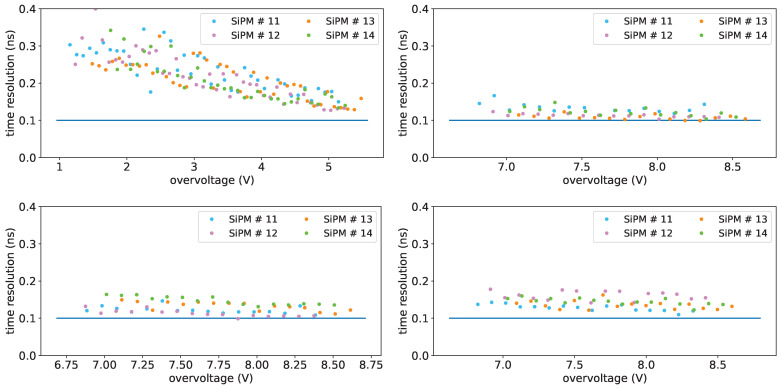
Single-photon time sigma vs. overvoltage for FBK $1\times 1\;{\mathrm{mm}}^{2}$ devices of set FBK1 at a temperature of −30 °C. Before irradiation on (**top left**), after first irradiation on (**bottom left**), after first annealing on (**top right**) and after second irradiation on (**bottom right**). Horizontal scales are not the same: after first data taking the overvoltage range was moved upwards. Horizontal line: reference for 100 ps.

Single-photon time sigma vs. overvoltage is shown in Figure 35 for the FBK $1\times 1\;{\mathrm{mm}}^{2}$ of set FBK1 at a temperature of −30 °C; the $3\times 3\;{\mathrm{mm}}^{2}$ from FBK, as previously stated, were not usable, while the Hamamatsu one has a poorer sigma around 300 ps, so it will not be discussed here.

From these measurements we get that, in order to reach the desired time resolution, these SiPMs must be operated with an overvoltage of 9 V or more.

**Figure 36:**
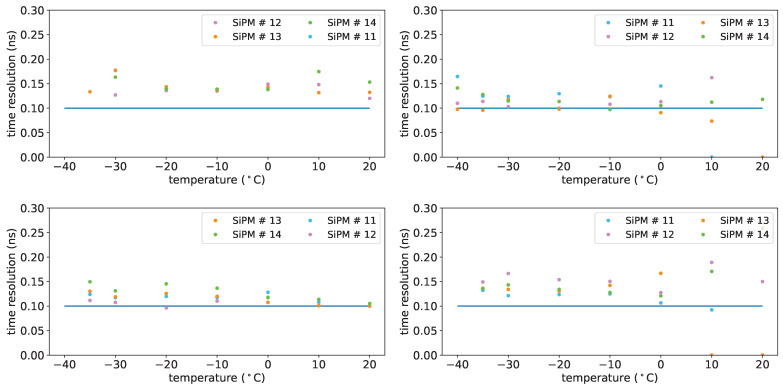
Single-photon time sigma for FBK $1\times 1\;{\mathrm{mm}}^{2}$ devices before irradiation (**top left**, overvoltage 5 V), after first irradiation (**top right**, overvoltage 8 V), after first annealing (**bottom left**, overvoltage 8 V) and after second irradiation (**bottom right**, overvoltage 8 V). Horizontal line: reference for 100 ps.

Figure 36 shows the single-photon time sigma as a function of the temperature before irradiation, at an overvoltage of 5 V, after irradiation, after annealing, after re-irradiation at an overvoltage of 8V.

The time sigma plots do not show significant worsening for irradiation with fluences up to $2\times 10^{10}$ neq/cm^2^ or for the annealing process.

Time resolution after re-irradiation is also found not significantly degraded. The 50% worsening described above concerns only fluence of $1\times 10^{10}$ neq/cm^2^ (SiPM #12), it may be due to a SiPM more sensitive than others to neutron damages.

### 4.6. Dark Count Rates

The dark count rate is the measure of the noise, in terms of photon signals, affecting the device; as we have seen this contributes to the degradation both of the amplitude and the timing of the signal.

To measure the dark count rate we take data with random triggers and count the number of signals above a threshold corresponding to half the amplitude of the single-photon one.

**Figure 37:**
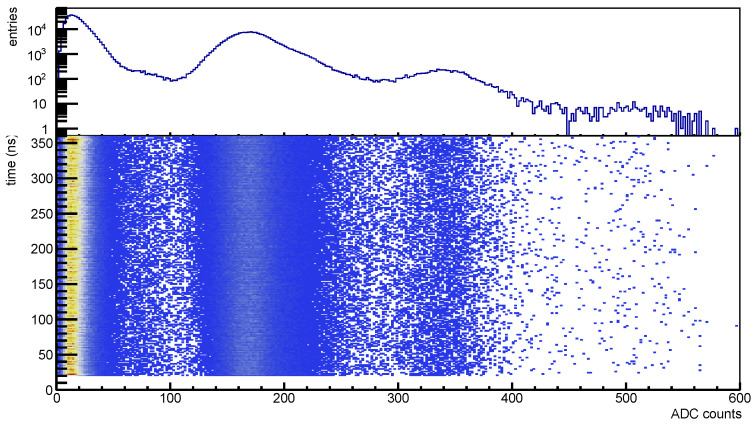
Dark count rate plots: on (**top**) amplitude spectra (ADC counts), on (**bottom**) the scatter plot of the signal time (ns) vs. the amplitude.

In Figure 37 a plot showing the time vs. the peak amplitude distribution for a dark count rate measurement; on top the amplitude distribution shows the noise peak plus peaks corresponding to single, two and three photons signals, on bottom the time-amplitude correlation from which we observe that the signals are uniformly distributed in time. This distribution refers to an irradiated device.

In order to reduce systematics we cut the edges of the time interval considered where the peak finding algorithm has some problems in dealing with the extremes of the spectra.

**Figure 38:**
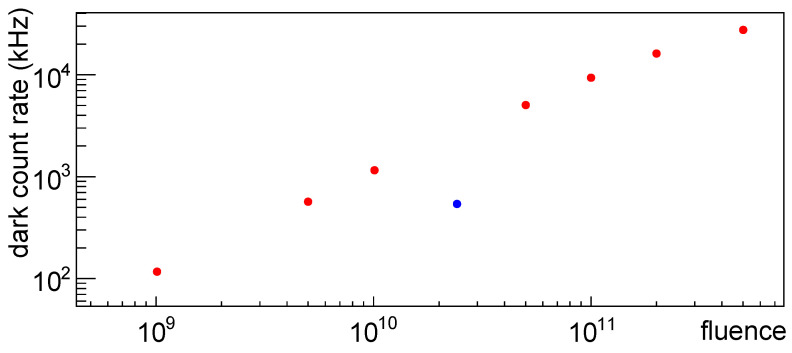
Dark count rate for SiPMs of set H1 at −20 °C and at an overvoltage of 2 V plotted vs. fluence of first irradiation. In blue DCR for SiPM #4, in red for the others.

In Figure 38 the dark count rate vs. fluence at a temperature of −20 °C and at 2 V of overvoltage is shown for set H1 after first irradiation. Given the fact that all the eight SiPMs, at an overvoltage of 2 V, had a dark count rate compatible among them, we can treat this data, as a first order approximation, as it was coming from the same device at various fluences. Both scales are logarithmic.

From this plot we can see that most of the points follow an almost linear distribution (in this scale), but there’s a point, marked in blue, that is apart from this distribution; this corresponds to SiPM #4 and it is present at all temperatures, so we can attribute this to some misbehavior of this device.

**Figure 39:**
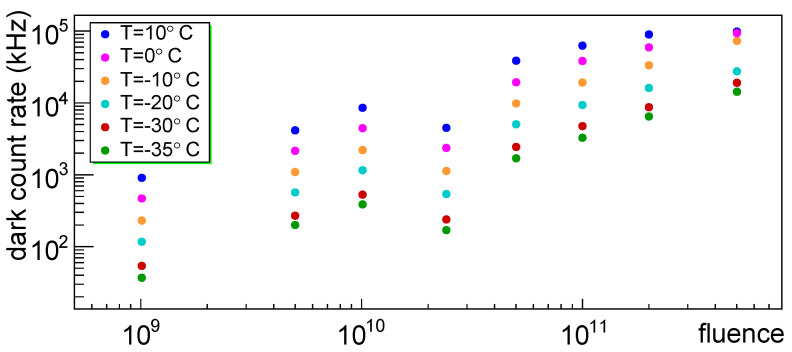
Dark count rate for SiPM set H1 plotted vs. fluence of first irradiation at various temperatures.

In Figure 39 the dark count rate vs. fluence is shown for different temperatures; we can notice that at higher temperatures the rates tend to saturate for the most irradiated devices. We can notice that SiPM #4 constantly tends to present lower rates.

**Figure 40:**
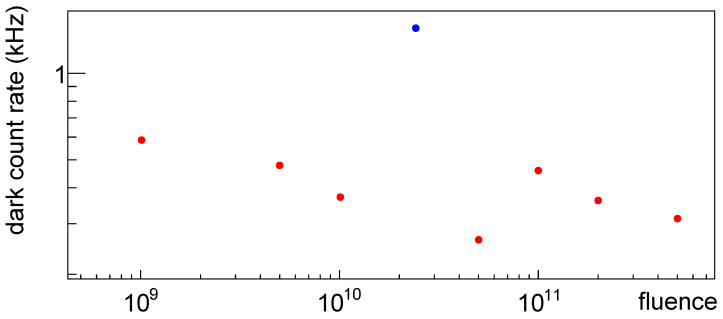
Dark count rate for SiPMs of set H1 plotted vs. fluence of first irradiation at −20 °C before irradiation at an overvoltage of 2 V. In blue DCR for SiPM #4, in red for the others.

In Figure 40 the dark count rate before irradiation is shown; on the *x*-axis the irradiation for the given device just for comparison with Figure 38 and Figure 39; what we can see is that before irradiation SiPM #4 showed a larger dark count rate, while after irradiation a lower one w.r.t. what one could expect. We noticed some other strange behavior of this device; further studies are needed to understand if with this measurement one can spot problematic devices.

**Figure 41:**
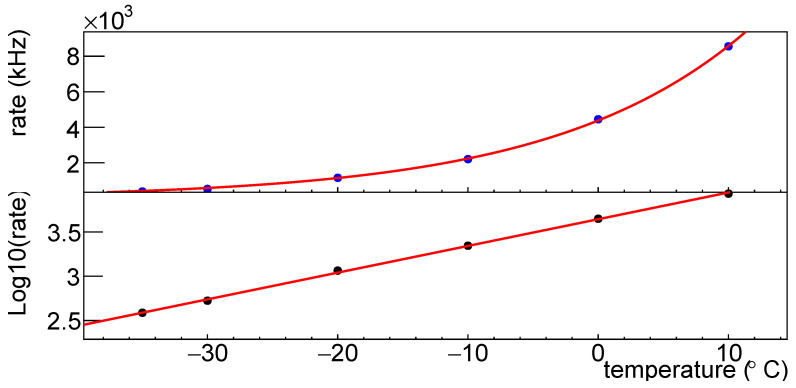
Dark count rate for SiPM #5 plotted vs. temperature after first irradiation at an overvoltage of 2 V. Rates in linear scale on top, in log10 scale on bottom.

We can also study the dependency on the temperature of the dark count rate; Figure 41 shows the dark count rate vs. temperature for SiPM #5, at an overvoltage of 2 V, after first irradiation (1.02× 10^10^ neq/cm^2^).

In the top plot the dark count rate vs. temperature is shown; this distribution is fitted with the function $r=N\cdot 10^{t\cdot a}$, where *N* is a normalization factor that depends on the irradiation/annealing, while *a* is a parameter that is always between 0.03 and 0.04${{(}^{\circ }C)}^{-1}$ for set H1 before and after irradiations and annealings. In the bottom plot the log10 of the dark count rate vs. temperature is shown: it is clearly visible a linear dependency with the temperature. Only for the SiPM irradiated at 5.07 × 10^11^ neq/cm^2^ the fit does not converge: the reason stands in the dark count rate saturation at higher temperatures. Also for $1\times 1\;{\mathrm{mm}}^{2}$ FBK1 SiPMs we see the same behavior; the exponent parameter is a little bit smaller, around 0.02.

**Figure 42:**
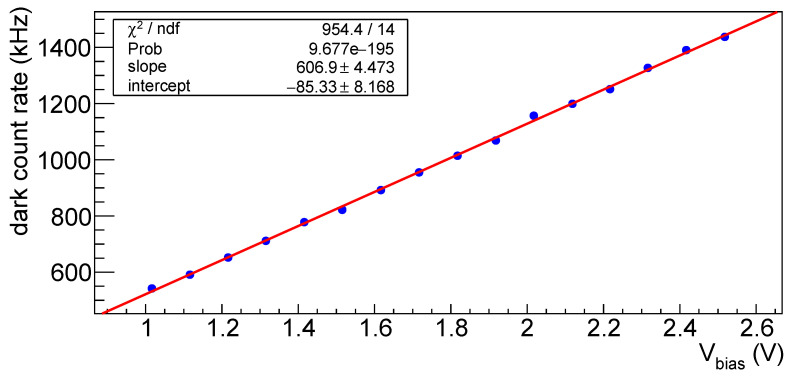
Dark count rate for SiPM #5 vs. overvoltage after first irradiation at t = −20 °C. In the box on top left of the plot the results of the linear fit are shown as an example.

In Figure 42 the dark count rate as a function of the overvoltage is shown for SiPM #5 at a temperature of −20 °C; a similar behavior is seen at different temperatures and for all SiPMs of set H1.

We have seen that time resolution can be improved by increasing the overvoltage, but one has also to consider the dark count rate increase with the bias voltage, as suggested by Figure 42. In Table 17 the variation of darkcount rate with the overvolatge is reported for H1 devices after first irradiation as a function of fluence at a temperature of −10 °C, while in Table 18 it is reported for SiPM #3 at different temperatures.

For $3\times 3\;{\mathrm{mm}}^{2}$ devices from FBK, Ketek and OnSemi we couldn’t define the single-photon amplitude, for this reason we don’t have dark count rate measurements for them.

## 5. Conclusions

From our studies we can conclude that, with the tested SiPMs, for a time resolution below 100 ps on the single photon the best choice is a small device ($1\times 1\;{\mathrm{mm}}^{2}$ or $1.3\times 1.3\;{\mathrm{mm}}^{2}$), with large cells. Radiation induced damaged can be partially cured by annealing with high temperatures (of the order of 150 °C).

The time resolution does not show significant worsening for irradiation with fluences up to $2\times 10^{10}$ neq/cm^2^, we may say that time resolution is affected by radiation damage only through the induced noise, mainly in terms of photon equivalent signals generated internally in the SiPM.

Dark count rate can be reduced by lowering the working temperature, but the effect is not linear and becomes less relevant at temperatures below −30 °C, at least for the tested devices, so a combination of annealing and lowering temperature is mandatory to operate irradiated devices.

We observed also some worsening of performances on some devices at low temperatures; this must be investigated with a large sample in a more systematic way.

We still have to conclude the analysis on the acquired data and we will study annealing with forward biasing.

## Figures and Tables

**Table 1:** SiPM sets.

Set	ID	Model	Set	ID	Model
H1	0	Hamamatsu S13360-1350PE	MIX1	16	Ketek PM3315-WL
	1	Hamamatsu S13360-1350PE		17	Ketek PM3315-WL
	2	Hamamatsu S13360-1350PE		18	Ketek PM3335-WL
	3	Hamamatsu S13360-1350PE		19	Ketek PM3335-WL
	4	Hamamatsu S13360-1350PE		20	OnSemi 10035
	5	Hamamatsu S13360-1350PE		21	OnSemi 10035
	6	Hamamatsu S13360-1350PE		22	OnSemi 30035
	7	Hamamatsu S13360-1350PE		23	OnSemi 30035
FBK1	8	FBK NUV-HD-RH-3015	H2	24	Hamamatsu S13360-3025PE
	9	FBK NUV-HD-RH-3015		25	Hamamatsu S13360-3025PE
	10	FBK NUV-HD-RH-3015		26	Hamamatsu S13360-3050PE
	11	FBK NUV-HD-RH-1015		27	Hamamatsu S13360-3050PE
	12	FBK NUV-HD-RH-1015		28	Hamamatsu S14160-3015PS
	13	FBK NUV-HD-RH-1015		29	Hamamatsu S14160-3015PS
	14	FBK NUV-HD-RH-1015		30	Hamamatsu S14160-3050HS
	15	Hamamatsu S14160-3050HS		31	Hamamatsu S14160-3050HS

**Table 2:** SiPM geometrical characteristics.

Model	Size (mm^2^)	Pixel (μm)
Hamamatsu S13360-1350PE	1.3×1.3	50
Hamamatsu S14160-3050HS	3×3	50
Hamamatsu S13360-3050PE	3×3	50
Hamamatsu S13360-3025PE	3×3	25
Hamamatsu S14160-3015PS	3×3	15
Ketek PM3315-WL	3×3	15
Ketek PM3335-WL	3×3	35
OnSemi 10035	1×1	35
OnSemi 30035	3×3	35
FBK NUV-HD-RH-1015	1×1	15
FBK NUV-HD-RH-3015	3×3	15

**Table 3:** Integrated neutron fluences (neq/cm^2^) for SiPMs of set H1 obtained in the two irradiation campaigns.

SiPM ID	November 2022	April 2024
0	5.07 × 10^11^	9.97 × 10^9^
1	2.03 × 10^11^	1.01 × 10^10^
2	1.01 × 10^11^	1.00 × 10^10^
3	5.07 × 10^10^	9.99 × 10^9^
4	2.45 × 10^10^	9.99 × 10^9^
5	1.02 × 10^10^	9.99 × 10^9^
6	5.06 × 10^9^	1.00 × 10^10^
7	1.03 × 10^9^	1.00 × 10^10^

**Table 4:** Integrated neutron fluences (neq/cm^2^) for SiPMs of set FBK1 obtained in the two irradiation campaigns.

SiPM ID	July 2023	April 2024
8	1.01 × 10^10^	9.99 × 10^9^
9	4.98 × 10^9^	1.00 × 10^10^
10	1.01 × 10^9^	1.00 × 10^10^
11	2.00 × 10^10^	9.99 × 10^9^
12	1.00 × 10^10^	9.99 × 10^9^
13	5.00 × 10^9^	1.00 × 10^10^
14	1.04 × 10^9^	1.00 × 10^10^
15	9.94 × 10^8^	1.00 × 10^10^

**Table 5:** Integrated neutron fluences (neq/cm^2^) for SiPMs of set MIX1 obtained in the two irradiation campaigns.

SiPM ID	July 2023	April 2024
16	1.00 × 10^10^	1.01 × 10^10^
17	1.03 × 10^9^	1.00 × 10^10^
18	1.00 × 10^10^	9.99 × 10^9^
19	1.00 × 10^9^	1.00 × 10^10^
20	1.00 × 10^10^	9.99 × 10^9^
21	1.12 × 10^9^	1.00 × 10^10^
22	1.00 × 10^10^	9.98 × 10^9^
23	1.05 × 10^9^	1.00 × 10^10^

**Table 6:** Breakdown voltage (V) before first irradiation for SiPMs of set H1.

T (°C)	SiPM # 0	SiPM # 1	SiPM # 2	SiPM # 3	SiPM # 4	SiPM # 5	SiPM # 6	SiPM # 7
20	51.54 ± 0.14	51.39 ± 0.24	51.14 ± 0.30	51.26 ± 0.23	51.12 ± 0.14	51.22 ± 0.22	51.32 ± 0.19	51.62 ± 0.10
15	51.24 ± 0.09	51.07 ± 0.09	50.76 ± 0.09	50.94 ± 0.07	50.80 ± 0.08	50.93 ± 0.05	51.01 ± 0.12	51.34 ± 0.12
10	50.94 ± 0.09	50.81 ± 0.29	50.51 ± 0.10	50.66 ± 0.12	50.51 ± 0.08	50.63 ± 0.11	50.74 ± 0.10	51.05 ± 0.04
0	50.42 ± 0.08	50.29 ± 0.04	49.97 ± 0.07	50.15 ± 0.03	50.02 ± 0.14	50.13 ± 0.06	50.22 ± 0.02	50.52 ± 0.06
−10	49.84 ± 0.11	49.70 ± 0.07	49.38 ± 0.08	49.57 ± 0.06	49.44 ± 0.15	49.54 ± 0.06	49.63 ± 0.07	49.93 ± 0.08
−20	49.29 ± 0.04	49.16 ± 0.05	48.85 ± 0.06	49.03 ± 0.03	48.90 ± 0.08	49.00 ± 0.01	49.09 ± 0.01	49.38 ± 0.05
−30	48.73 ± 0.03	48.60 ± 0.10	48.29 ± 0.01	48.46 ± 0.05	48.29 ± 0.07	48.44 ± 0.03	48.52 ± 0.03	48.82 ± 0.03
−35	48.44 ± 0.08	48.32 ± 0.02	48.00 ± 0.02	48.19 ± 0.02	48.05 ± 0.04	48.15 ± 0.05	48.24 ± 0.03	48.53 ± 0.04
−40	48.16 ± 0.07	48.05 ± 0.04	47.73 ± 0.02	47.91 ± 0.03	-	-	47.96 ± 0.03	48.27 ± 0.03

**Table 7:** Gain variation over bias voltage (mV/V) before first irradiation for SiPMS of set H1.

T (°C)	SiPM # 0	SiPM # 1	SiPM # 2	SiPM # 3	SiPM # 4	SiPM # 5	SiPM # 6	SiPM # 7
20	16.66 ± 0.03	16.58 ± 0.05	17.64 ± 0.07	18.24 ± 0.06	16.89 ± 0.03	17.66 ± 0.05	17.56 ± 0.05	17.69 ± 0.02
15	16.46 ± 0.02	16.15 ± 0.02	16.91 ± 0.02	17.86 ± 0.02	16.57 ± 0.02	17.50 ± 0.01	17.32 ± 0.03	17.65 ± 0.03
10	16.30 ± 0.02	16.33 ± 0.07	16.99 ± 0.02	17.83 ± 0.03	16.47 ± 0.02	17.40 ± 0.03	17.42 ± 0.02	17.51 ± 0.01
0	16.36 ± 0.02	16.36 ± 0.01	17.09 ± 0.02	18.02 ± 0.01	16.88 ± 0.03	17.71 ± 0.01	17.60 ± 0.00	17.71 ± 0.01
−10	16.29 ± 0.02	16.25 ± 0.02	17.02 ± 0.02	17.87 ± 0.02	16.82 ± 0.03	17.59 ± 0.03	17.60 ± 0.02	17.58 ± 0.02
−20	16.31 ± 0.01	16.15 ± 0.01	17.12 ± 0.01	17.85 ± 0.01	16.77 ± 0.02	17.48 ± 0.00	17.57 ± 0.00	17.53 ± 0.01
−30	16.23 ± 0.01	16.14 ± 0.02	17.14 ± 0.00	17.78 ± 0.01	16.52 ± 0.02	17.44 ± 0.01	17.43 ± 0.01	17.50 ± 0.01
−35	16.13 ± 0.02	16.03 ± 0.01	16.99 ± 0.00	17.71 ± 0.01	16.62 ± 0.01	17.28 ± 0.01	17.37 ± 0.01	17.33 ± 0.01
−40	16.11 ± 0.02	15.96 ± 0.01	16.98 ± 0.00	17.62 ± 0.0	-	-	17.30 ± 0.01	17.31 ± 0.02

**Table 8:** Thermal coefficient of breakdown voltage before irradiation. The average value is 0.05588±0.00007V/°C.

SiPM #	Slope (V/°C)
0	0.05585 ± 0.00019
1	0.05567 ± 0.00025
2	0.05578 ± 0.00028
3	0.05581 ± 0.00023
4	0.05533 ± 0.00040
5	0.05550 ± 0.00023
6	0.05630 ± 0.00015
7	0.05583 ± 0.00016

**Table 9:** Breakdown voltage (V) before first irradiation, set FBK1. It was not possible to extract any useful data from the $3\times 3\;{\mathrm{mm}}^{2}$ FBK SiPMs.

T (°C)	SiPM # 11	SiPM # 12	SiPM # 13	SiPM # 14	SiPM # 15
20	-	32.44 ± 0.04	32.12 ± 0.06	32.33 ± 0.06	37.94 ± 0.03
10	-	32.11 ± 0.03	31.87 ± 0.06	32.03 ± 0.06	37.54 ± 0.04
0	-	31.77 ± 0.03	31.55 ± 0.03	31.72 ± 0.04	37.15 ± 0.11
−10	31.55 ± 0.08	31.45 ± 0.02	31.21 ± 0.10	31.42 ± 0.04	36.76 ± 0.01
−20	-	31.11 ± 0.02	30.94 ± 0.10	31.08 ± 0.04	36.39 ± 0.05
−30	30.85 ± 0.07	30.79 ± 0.03	30.55 ± 0.06	30.78 ± 0.06	36.10 ± 0.05
−35	30.76 ± 0.18	30.62 ± 0.01	30.43 ± 0.10	30.61 ± 0.02	35.88 ± 0.04
−40	30.81 ± 0.19	30.46 ± 0.02	30.78 ± 0.17	30.54 ± 0.54	35.66 ± 0.04

**Table 10:** Gain variation over bias voltage (mV/V) before first irradiation for set FBK1.

T (°C)	SiPM # 11	SiPM # 12	SiPM # 13	SiPM # 14	SiPM # 15
20	-	4.308 ± 0.020	4.210 ± 0.009	4.054 ± 0.009	6.195 ± 0.014
10	-	4.270 ± 0.015	4.118 ± 0.009	4.038 ± 0.012	6.394 ± 0.016
0	-	4.105 ± 0.031	4.149 ± 0.010	4.013 ± 0.008	6.443 ± 0.024
−10	3.279 ± 0.005	4.085 ± 0.011	3.984 ± 0.011	3.944 ± 0.014	6.226 ± 0.027
−20	-	4.100 ± 0.022	3.993 ± 0.010	3.925 ± 0.011	6.342 ± 0.014
−30	3.924 ± 0.006	4.144 ± 0.008	4.076 ± 0.003	4.040 ± 0.008	6.325 ± 0.011
−35	3.89 ± 0.01	4.083 ± 0.016	3.998 ± 0.004	3.974 ± 0.007	6.261 ± 0.018
−40	4.10 ± 0.02	3.906 ± 0.015	3.829 ± 0.014	3.844 ± 0.009	6.101 ± 0.023

**Table 11:** Breakdown voltage (V) after first irradiation, set FBK1.

T (°C)	SiPM # 11	SiPM # 12	SiPM # 13	SiPM # 14	SiPM # 15
20	32.58 ± 1.53	31.85 ± 0.25	31.83 ± 0.08	32.12 ± 0.10	-
10	31.45 ± 0.54	31.77 ± 0.13	31.66 ± 0.03	31.81 ± 0.06	37.38 ± 0.25
0	26.10 ± 8.80	31.32 ± 0.21	31.19 ± 0.25	31.43 ± 0.13	37.00 ± 0.13
−10	31.45 ± 0.13	31.28 ± 0.22	30.93 ± 0.07	31.11 ± 0.03	36.67 ± 0.05
−20	31.05 ± 0.04	30.80 ± 0.06	30.66 ± 0.07	30.82 ± 0.03	36.34 ± 0.05
−30	30.70 ± 0.09	30.56 ± 0.11	30.43 ± 0.04	30.46 ± 0.08	35.96 ± 0.06
−35	30.49 ± 0.07	30.43 ± 0.08	30.31 ± 0.06	30.23 ± 0.19	35.79 ± 0.10
−40	30.39 ± 0.19	30.22 ± 0.13	30.20 ± 0.14	30.23 ± 0.08	35.67 ± 0.17

**Table 12:** Breakdown voltage (V) after first annealing, set FBK1.

T (°C)	SiPM # 11	SiPM # 12	SiPM # 13	SiPM # 14	SiPM # 15
20	32.32 ± 0.14	32.18 ± 0.04	32.07 ± 0.10	32.12 ± 0.07	38.09 ± 0.19
10	31.95 ± 0.06	31.84 ± 0.02	31.59 ± 0.07	31.82 ± 0.08	37.67 ± 0.10
0	31.60 ± 0.03	31.52 ± 0.04	31.26 ± 0.01	31.45 ± 0.07	37.25 ± 0.12
−10	31.24 ± 0.04	31.10 ± 0.24	30.99 ± 0.04	31.12 ± 0.11	36.88 ± 0.06
−20	30.94 ± 0.08	30.83 ± 0.02	30.64 ± 0.06	30.82 ± 0.04	36.60 ± 0.10
−30	30.63 ± 0.05	30.64 ± 0.09	30.42 ± 0.14	30.52 ± 0.07	36.31 ± 0.17
−35	30.38 ± 0.17	30.44 ± 0.34	30.30 ± 0.05	30.42 ± 0.12	36.22 ± 0.41

**Table 13:** Breakdown voltage (V) after second irradiation, set FBK1.

T (°C)	SiPM # 11	SiPM # 12	SiPM # 13	SiPM # 14	SiPM # 15
20	31.88 ± 0.37	31.07 ± 0.22	31.65 ± 0.05	31.45 ± 0.64	-
10	31.51 ± 0.56	31.42 ± 0.21	31.49 ± 0.06	31.76 ± 0.10	-
0	31.48 ± 0.11	31.46 ± 0.08	31.37 ± 0.10	31.37 ± 0.16	-
−10	31.12 ± 0.30	31.07 ± 0.07	30.90 ± 0.10	31.06 ± 0.02	-
−20	30.95 ± 0.03	31.06 ± 0.25	30.62 ± 0.13	30.80 ± 0.08	-
−30	30.67 ± 0.01	30.58 ± 0.08	30.40 ± 0.09	30.43 ± 0.05	-
−35	30.39 ± 0.22	30.47 ± 0.08	30.37 ± 0.14	30.33 ± 0.08	36.02 ± 0.49

**Table 14:** Breakdown voltage (V) before and after first irradiation for SiPMs # 1, 3, 5 and 7 (see Table 3) for irradiation levels).

	SiPM # 1		SiPM # 3		SiPM # 5		SiPM # 7	
T (°C)	Before	Irradiated	Before	Irradiated	Before	Irradiated	Before	Irradiated
10	50.81 ± 0.29	-	50.66 ± 0.12	51.12 ± 2.16	50.63 ± 0.11	50.71 ± 0.15	51.05 ± 0.04	51.09 ± 0.14
0	50.29 ± 0.04	-	50.15 ± 0.03	50.22 ± 0.13	50.13 ± 0.06	50.12 ± 0.12	50.52 ± 0.06	50.49 ± 0.08
−10	49.70 ± 0.07	-	49.57 ± 0.06	49.62 ± 0.10	49.54 ± 0.06	49.55 ± 0.13	49.93 ± 0.08	49.92 ± 0.14
−20	49.16 ± 0.05	48.81 ± 1.93	49.03 ± 0.03	49.02 ± 0.06	49.00 ± 0.01	48.96 ± 0.09	49.38 ± 0.05	49.34 ± 0.07
−30	48.60 ± 0.10	48.55 ± 0.36	48.46 ± 0.05	48.45 ± 0.16	48.44 ± 0.03	48.43 ± 0.03	48.82 ± 0.03	48.78 ± 0.08
−35	48.32 ± 0.02	48.31 ± 0.29	48.19 ± 0.02	48.20 ± 0.10	48.15 ± 0.05	48.15 ± 0.10	48.53 ± 0.04	48.51 ± 0.06

**Table 15:** Breakdown voltage (V) before first irradiation, set H2.

T (°C)	SiPM # 24	SiPM # 25	SiPM # 26	SiPM # 27	SiPM # 28	SiPM # 29	SiPM # 30	SiPM # 31
20	51.01 ± 0.04	51.42 ± 0.64	51.72 ± 0.01	51.75 ± 0.02	37.62 ± 1.02	27.71 ± 102.27	37.83 ± 0.07	38.41 ± 0.78
10	50.24 ± 0.15	50.88 ± 1.70	51.13 ± 0.09	51.16 ± 0.07	36.95 ± 1.28	33.95 ± 17.12	37.45 ± 0.03	37.92 ± 0.07
0	49.72 ± 0.15	49.98 ± 0.31	50.51 ± 0.02	50.57 ± 0.04	36.80 ± 0.85	39.58 ± 2.05	37.04 ± 0.04	37.51 ± 0.06
−10	49.03 ± 0.40	51.66 ± 1.86	49.94 ± 0.01	50.04 ± 0.34	38.06 ± 0.57	38.30 ± 0.49	36.68 ± 0.05	37.10 ± 0.05
−20	48.59 ± 0.30	50.06 ± 1.55	49.35 ± 0.06	49.43 ± 0.12	37.16 ± 0.40	38.06 ± 0.46	36.23 ± 0.04	37.03 ± 0.33
−30	48.40 ± 0.61	48.94 ± 1.30	48.76 ± 0.08	48.80 ± 0.05	37.06 ± 0.34	42.83 ± 10.59	35.87 ± 0.04	36.29 ± 0.26
−35	47.90 ± 0.32	48.10 ± 4.37	48.46 ± 0.07	48.55 ± 0.05	36.59 ± 0.75	42.84 ± 8.16	35.66 ± 0.03	36.08 ± 0.07

**Table 16:** Gain variation over bias voltage (mV/V) before first irradiation for set H2.

T	SiPM # 24	SiPM # 25	SiPM # 26	SiPM # 27	SiPM # 28	SiPM # 29	SiPM # 30	SiPM # 31
20	1.920 ± 0.001	1.989 ± 0.016	6.249 ± 0.001	6.550 ± 0.001	0.675 ± 0.012	0.265 ± 0.510	6.240 ± 0.008	3.260 ± 0.045
10	1.870 ± 0.004	2.009 ± 0.044	6.244 ± 0.007	6.530 ± 0.006	0.655 ± 0.014	0.452 ± 0.136	6.233 ± 0.003	5.590 ± 0.007
0	1.872 ± 0.004	1.889 ± 0.008	6.202 ± 0.001	6.523 ± 0.003	0.668 ± 0.010	0.976 ± 0.033	6.208 ± 0.004	5.475 ± 0.006
−10	1.844 ± 0.010	2.416 ± 0.058	6.197 ± 0.001	6.557 ± 0.030	0.792 ± 0.007	0.823 ± 0.007	6.216 ± 0.006	3.295 ± 0.003
−20	1.862 ± 0.007	2.124 ± 0.043	6.181 ± 0.005	6.504 ± 0.011	0.740 ± 0.005	0.825 ± 0.006	6.121 ± 0.004	5.804 ± 0.034
−30	1.916 ± 0.015	2.008 ± 0.035	6.144 ± 0.007	6.421 ± 0.005	0.760 ± 0.004	2.261 ± 0.387	6.112 ± 0.004	5.489 ± 0.026
−35	1.878 ± 0.008	1.886 ± 0.110	6.121 ± 0.006	6.426 ± 0.004	0.732 ± 0.009	2.197 ± 0.288	6.075 ± 0.003	5.481 ± 0.007

**Table 17:** Variation of darkcount rate for SiPMs of set H1 with irradiation after first campaign at −10 °C.

Fluence (neq/cm^2^)	DCR Variation (kHz/V)
5.07 × 10^11^	32.8 × 10^3^
2.03 × 10^11^	17.2 × 10^3^
1.01 × 10^11^	9.70 × 10^3^
5.07 × 10^10^	5.14 × 10^3^
2.45 × 10^10^	0.62 × 10^3^
1.02 × 10^10^	1.21 × 10^3^
5.06 × 10^9^	0.60 × 10^3^
1.03 × 10^9^	0.13 × 10^3^

**Table 18:** Variation of darkcount rate for SiPM #3 of set H1 after first irradiation.

Temperature (°C)	DCR Variation (kHz/V)
10	17.4 × 10^3^
0	9.6 × 10^3^
−10	5.1 × 10^3^
−20	2.6 × 10^3^
−30	1.4 × 10^3^
−35	0.9 × 10^3^

## Data Availability

The original contributions presented in this study are included in the article, and further inquiries can be directed to the corresponding author.

## Abbreviations

The following abbreviations are used in this manuscript:

SiPM	silicon photomultiplier
FBK	Fondazione Bruno Kessler
